# The effectiveness of health education programs on the Opisthorchi viverrini in junior high school, Nakhon Ratchasima, Thailand

**DOI:** 10.1186/2047-2994-4-S1-P115

**Published:** 2015-06-16

**Authors:** S Kaewpitoon, N Kaewpitoon

**Affiliations:** 1 Family Medicine and Community Medicine, Suranaree University of Technology, Nakhon Ratchasima, Thailand; 2 Public Health, Vongchavalitkul University, Nakhon Ratchasima, Thailand

## Introduction

*Opisthorchis viverrini* is a major public health problem in Thailand. It is associated with cholangiocarcinoma, the highly fatal bile duct cancer. Therefore, experimental research was conducted to improve the knowledge on the *Opisthorchis viverrini* using health education program among junior high school in Nakon Ratchasima province Thailand.

## Objectives

Experimental research was conducted to improve the knowledge on the *Opisthorchis viverrini* using health education program among junior high school in Nakon Ratchasima province Thailand during November 2010 and January 2011.

## Methods

Health education programs were created as follows a short movie, pamphlet, and game related to *O. viverrini* knowledge and perception. 200 students (12 and 15 years old) in secondary school were studied. The students were assigned an experimental (150 students) and control (50 students) group. The experimental group participated in the designated program activities for 2 weeks. Student knowledge levels were collected by questionnaires before and after the intervention program.

## Results

The results indicate that the experimental group had significantly increased its knowledge of *O. viverrini* (126/150 students, 84%), perceptions of disease transmission, severity, prevention and control (p<0.01). It was also found that knowledge of *O. viverrini* transmission, severity, prevention and control, were significantly correlated with health education programs (p<0.001). In addition, the highly attractive and effectiveness were found in the short movie and game more than pamphlet (p<0.001).

## Conclusion

The studied suggested that health education programs were reach and effective to improve student knowledge particularly short movie and game. Moreover, this attractive movie and game could be realized the *O. viverrini* transmission, severity, prevention and control, it is recommended that this health education programs should be applied to other similar school to *O. viverrini* prevent and control in new generation age of Thailand.

## Disclosure of interest

None declared.
